# Optimization of microalgal growth parameters for enhanced biohydrogen production via biophotolysis in *Chlorella* sp.

**DOI:** 10.1007/s10529-026-03734-4

**Published:** 2026-04-30

**Authors:** Yi Jie Liew, Sie Yon Lau, Murat Yılmaz, Man Kee Lam, Jun Wei Lim

**Affiliations:** 1 Chemical and Energy Engineering, Faculty of Engineering and Science, Curtin University Malaysia, CDT 250, 98009 Miri, Sarawak Malaysia; 2 Department of Chemistry and Chemical Processing Technologies, Bahçe Vocational School, Osmaniye Korkut Ata University, 80000 Osmaniye, Türkiye; 3 Chemical Engineering Department, Universiti Teknologi Petronas, 32610 Seri Iskandar, Perak Darul Ridzuan Malaysia; 4 HICoE-Centre for Biofuel and Biochemical Research, Institute of Self-Sustainable Building, Universiti Teknologi Petronas, 32610 Seri Iskandar, Perak Malaysia

**Keywords:** Biohydrogen, Microalgae, Biophotolysis, Cell growth, Nitrogen purging, Photoperiod, Carbon source

## Abstract

The increasing reliance on fossil fuels for global energy production has intensified greenhouse gas emissions, highlighting the need for sustainable energy alternatives. Hydrogen is considered a promising green fuel due to its high energy density and conversion efficiency. Among various production pathways, microalgae-based biohydrogen generation via biophotolysis is particularly attractive owing to its high biomass productivity, adaptability to diverse water sources, flue gas mitigation potential, and low land requirements.This study investigates the effects of key microalgal growth parameters on biohydrogen production by *Chlorella* sp. through biophotolysis. The impacts of nitrogen purging during the transition from aerobic to anaerobic conditions, different photoperiod regimes (continuous illumination, continuous darkness, and a light–dark cycle), glucose supplementation (5, 10, and 15 g L⁻^1^), and temperature (25, 30, and 35 °C) were systematically evaluated. Microalgal cell density was monitored during hydrogen production to elucidate its relationship with hydrogen yield. Initial experiments were conducted in 10 mL test tubes to identify optimal conditions, which were subsequently applied to scale-up experiments in a 1000 mL jacketed reactor. Nitrogen purging significantly enhanced hydrogen production by removing oxygen and activating hydrogenase, resulting in a peak hydrogen concentration of 11 ppm. Continuous illumination yielded higher hydrogen levels than darkness and light–dark cycling. Glucose addition substantially increased hydrogen production, with the highest yield observed at 15 g L⁻^1^ (30 ppm). An optimal temperature of 30 °C also maximized hydrogen production. Under these conditions, hydrogen production increased as cell density decreased due to metabolic shifts. Scale-up experiments achieved a 405-fold increase in hydrogen yield, demonstrating the scalability potential of the process. These findings emphasize the importance of optimizing algal growth conditions to balance microalgal growth and biohydrogen production for future industrial applications.

## Introduction

Fossil fuels continue to dominate global energy production, accounting for approximately 75% of worldwide energy consumption while constituting the major source of greenhouse gas (GHG) emissions, particularly carbon dioxide (CO₂). In this context, hydrogen has emerged as the most promising green fuel due to its exceptionally high gravimetric energy density of approximately 120–140 MJ kg⁻^1^, exceeding that of conventional fuels such as methane or natural gas (55.5 MJ kg^−1^) by up to 2.5 times; coal (24 MJ kg^−1^), diesel (48 MJ kg^−1^), liquefied petroleum gas (46.4 MJ kg^−1^), kerosene (46 MJ kg ^−1^), and gasoline (44.4 MJ kg^−1^) as much as 5.9 times (Sharma et al. 2021; Silva et al. 2024). Hydrogen combustion produces only water as a by-product, with no carbon monoxide, carbon dioxide, sulfur oxides, or nitrogen oxides (Mandotra et al. 2021). Hydrogen is widely used in fuel cells for transportation, aerospace, and electricity generation, as well as in industrial applications such as ammonia synthesis, methanol production, and iron and steel manufacturing (Mandotra et al. 2021; Ahmed et al. 2021). Projections indicate hydrogen could supply up to 8–10% of global energy demand by 2025, with long-term targets aiming to increase its share to approximately 18% by 2030 while significantly reducing production and delivery costs to 1.80 USD kg^−1^ (Sharma et al. 2021).

Currently, most industrial hydrogen is produced through fossil fuel reforming, which requires carbon capture and storage technologies for carbon neutrality (Sharma et al. 2021). Consequently, there is growing interest in biomass-derived hydrogen production pathways that offer waste-to-energy potential, reduced CO₂ emissions, and enhanced sustainability (Ahmed et al. 2021). Among biological routes, biohydrogen production using microorganisms such as cyanobacteria, fermentative bacteria, and microalgae has gained increasing attention. Microalgae are considered highly promising due to their rapid growth rates, high biomass and lipid productivity, ability to utilize flue gases, adaptability to diverse water sources, and minimal competition with arable land (Anwar et al. 2019; Li et al. 2022). Furthermore, microalgae can double their biomass in as little as 3.5 h, and 1 kg of dry algal biomass captures approximately 1.83 kg of CO_2_ (Anwar et al. 2019).

Biohydrogen production from microalgae proceeds through several metabolic pathways, which are classified as light-dependent mechanisms, including direct biophotolysis, indirect biophotolysis, and photo-fermentation, and light-independent dark fermentation. In direct biophotolysis, light energy drives water-splitting reactions, generating electrons that are subsequently used for hydrogen evolution. Hydrogen production through direct biophotolysis typically yields 1.2–73.5 mL L^−1^, with production periods of 5–10 days (Sharma et al. 2021). This pathway can produce hydrogen with high purity of up to 98%; however, its practical efficiency remains limited due to the simultaneous oxygen evolution, which rapidly inhibits the oxygen-sensitive [FeFe]-hydrogenase enzyme responsible for proton reduction (Rashid et al. 2013; Nagarajan et al. 2017; Ahmed et al. 2021; Mandotra et al. 2021). As a result, hydrogen production via direct biophotolysis is transient and difficult to sustain. Indirect biophotolysis offers a viable alternative by temporally separating photosynthesis and hydrogen evolution across two stages: aerobic biomass accumulation through photosynthetic CO₂ fixation, followed by anaerobic hydrogen production using stored organic compounds (Rashid et al. 2013; Li et al. 2022). During the initial aerobic phase, microalgae fix CO_2_ photosynthetically to accumulate carbohydrate reserves such as starch; subsequently, under anaerobic conditions, these endogenous substrates are metabolized to generate reducing equivalents that feed into hydrogenase-mediated hydrogen evolution (Rashid et al. 2013; El-Dalatony et al. 2020; Grechanik & Tsygankov 2022). This two-stage strategy yields substantially higher hydrogen production of 6.625–243 mL L^−1^, with production periods of 148–168 h (Sharma et al. 2021). Nevertheless, its practical application remains constrained by complex metabolic regulation, substrate competition, and sensitivity to environmental conditions, which collectively limit scalability (Ahmed et al. 2021).

Algal growth conditions profoundly influence both hydrogenase activity and the metabolic switching between photosynthetic growth and hydrogen-producing states. Oxygen concentration represents a critical determinant, as the enzyme’s sensitivity necessitates strict anaerobiosis for sustained hydrogen evolution (Touloupakis et al. 2021). Nitrogen purging effectively displaces dissolved oxygen from the culture medium, creating the reducing environment required for the activation of oxygen-sensitive hydrogenase enzymes and enabling sustained hydrogen production (Maturi et al. 2026). Previous studies have demonstrated that nitrogen purging increases 50% hydrogen yield from *Clostridium* sp. cultures when sparging with nitrogen (1.43 ± 0.12 mol mol^−1^ glucose) compared to non-sparged systems (0.85 ± 0.32 mol mol^−1^ glucose), highlighting the importance of oxygen removal (Mizuno et al. 2000).

Light availability and photoperiod critically influence photosynthetic activity, substrate accumulation, and hydrogenase activation. Continuous illumination has been shown to sustain photosynthetic electron flow and enhance hydrogen production once anaerobic conditions are established (Javed et al. 2022; Grechanik and Tsygankov 2022). Rashid et al. (2011) observed that *Chlorella vulgaris* produced 496 mL H_2_ L^−1^ over 47 h under fully illuminated conditions, compared to 348 mL H_2_ L^−1^ over 45 h in complete darkness, confirming light dependency for sustained hydrogenase activity. Gabrielyan et al. (2017) reported that *P. kessleri* RA-002 under a 24-h light/48-h dark cycle achieved 2.5-fold higher hydrogen yields than under continuous illumination.

Exogenous carbon sources such as glucose can enhance hydrogen yield by supporting heterotrophic metabolism, although economic feasibility remains a concern at larger scales (Rashid et al. 2011). Rashid et al. (2013) demonstrated maximum hydrogen production of 812 ± 5 mL L^−1^ from *Chlorella vulgaris* with 5 g L^−1^ glucose, while higher and lower concentrations yielded less. Jiménez-Llanos et al. (2020) optimized *Chlorella* sp. hydrogen production with 10 g L^−1^ glucose, achieving 2.85 mol mol^−1^ glucose. Temperature also plays a crucial role by regulating enzymatic activity, metabolic flux, and growth kinetics, with optimal ranges varying among microalgal species, where optimal temperature of mesophilic microalgae ranges from 25 to 40 ℃ (Javed et al. 2022). Javed et al. (2022) reported hydrogen production rates of 183 and 238 mL L^−1^ h^−1^ for *Chlorella* sp. at 37 and 40 ℃, respectively.

Despite the various advantages of bioprocessing techniques, biohydrogen production remains commercially unviable due to low yields (Javed et al. 2022; Rathi et al. 2023). This study systematically optimises algal growth parameters governing indirect biophotolysis in *Chlorella* sp. by evaluating the impacts of (i) nitrogen purging, (ii) photoperiod regimes, (iii) glucose supplementation concentration, and (iv) temperature to examine the relationship between biomass development and biohydrogen production. Furthermore, a scale-up study is conducted using a 1000 mL jacketed reactor to validate the optimal parameters identified at a laboratory scale, providing critical insights for the development of sustainable and scalable microalgal biohydrogen production strategies.

## Experimental

###  Materials

The microalgal strain (*Chlorella* sp.) employed in this study was isolated from a freshwater lake located at Curtin University, Sarawak, Malaysia. Algae culture broth (ACB; Sigma-Aldrich) was used as the growth medium for microalgae cultivation to provide essential nutrients, which consist of 1 g/L sodium nitrate, 0.513 g/L magnesium sulfate, 0.25 g/L dipotassium phosphate, 0.058 g/L calcium chloride, 0.05 g/L ammonium chloride, and 0.003 g/L ferric chloride (MilliporeSigma 2018). D( +)-glucose monohydrate (Merck, Germany) was used as an external organic carbon source to support microalgal metabolism and sustain hydrogen production under anaerobic conditions.

###  Calibration of cell concentration

Seven *Chlorella sp.* samples with different cell concentrations were prepared by serial dilution to establish a correlation between cell density and optical density (OD). A twofold serial dilution (dilution factor of 2) was applied to obtain seven samples spanning a range of cell concentrations from 1.800 × 10^6^ total cells/mL to 61.025 × 10^6^ total cells/mL. The optical density of each diluted sample was measured using a UV–Vis spectrophotometer (Lambda 25, double-beam; PerkinElmer) at a wavelength of 683 nm (Gonçalves et al. 2013).

**Fig. 1:**
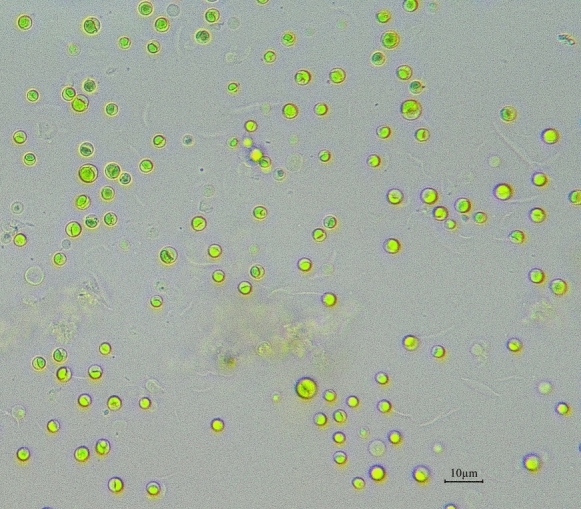
Optical image of microalgae cells on a hemocytometer under a microscope with 40 × magnification

To ensure accurate cell enumeration and minimize cell overlap, each sample was subsequently diluted tenfold before microscopic analysis. Microalgal cell counts were determined by pipetting 10 µL of each dilution onto a Neubauer hemocytometer (Improved XB.K.25, 0.10 mm depth, 1/400 mm^2^ grid; Qiujing) and covering with a coverslip. After a brief settling period, cells within the four corner squares were counted at 10 × magnification using an optical microscope equipped with a digital imaging system (Nikon Eclipse LV100N Pol). To ensure consistency and prevent double-counting, cells overlapping the exterior lines were only counted if they were touching the top or left-hand boundary lines of each square. Figure 1 shows the optical image of microalgae cells on hemocytometer under microscope with 10 × magnification.

Cell concentration was calculated using Eq. (1) (Benavides Escobar et al. 2025). The resulting calibration curve was used to convert OD measurements to cell concentrations in subsequent experiments.

1 $$ {\mathrm{Total}}\;{\text{cells/mL  =  }}\frac{{{\mathrm{Total}}\;{\mathrm{cells}}{\mkern 1mu} \;{\mathrm{counted}} \times {\mathrm{Dilution}}\;{\mathrm{factor}} \times {\mathrm{10000}}\;{\mathrm{cells/mL}}}}{{{\mathrm{Number}}\;{\mathrm{of}}\;{\mathrm{squares}}\;{\mathrm{counted}}}} $$

The calculated cell concentration for each dilution was recorded and plotted against the corresponding optical density (OD) values to generate a calibration curve. This calibration will enable the rapid, non-destructive monitoring of culture growth to accurately infer the cell concentration from the curve.

###  Inoculation and preparation of microalgae samples

The *Chlorella* sp. culture was concentrated by centrifugation using a refrigerated centrifuge (Universal 320R, Hettich) operated at 4000 rpm for 4 min at room temperature in 50 mL centrifuge tubes to obtain a high-density biomass suitable for hydrogen production. After centrifugation, the supernatant was carefully removed, leaving approximately 10 mL in each tube.

The growth medium was prepared in 250 mL Erlenmeyer flasks by dissolving 0.374 g of ACB in 200 mL of distilled water, corresponding to a final dry weight concentration of 1.87 g L⁻^1^, with a final pH of 7.0 ± 0.2 at 25 ℃ (MilliporeSigma 2018). The prepared medium was sterilized by autoclaving at 121 °C for 20 min using an autoclave (HVE-50, Hirayama).

Subsequently, 50 mL of the concentrated microalgal suspension, pooled from five centrifuge tubes, was aseptically transferred into each Erlenmeyer flask containing the sterilized growth medium.

###  Biomass accumulation stage under aerobic conditions

The initial OD of the microalgal cultures was measured at 683 nm using a UV–Vis spectrophotometer and recorded as 2.6303 ± 0.01. The prepared cultures were then cultivated under aerobic conditions with continuous aeration using an aquarium air pump (Hailea ACO-9610, 10 W), and illumination using cool-white fluorescent lamps at an intensity of 7780 lx at room temperature (maintained at 25 ± 2 ℃) for 24 h under continuous photoperiod to promote photosynthetic biomass accumulation. The pH of the culture medium was maintained at 7.0 ± 0.2 throughout the cultivation period. Following the cultivation period, the optical density was remeasured and increased to 2.8516 ± 0.01, indicating successful biomass growth. Subsequently, the cultures were aliquoted and transferred into 10 mL test tubes for further experimental procedures.

###  Hydrogen production stage under anaerobic conditions

The experimental vessels were hermetically sealed with rubber stoppers to ensure anaerobic conditions throughout the hydrogen production stage (Rashid et al. 2009). For each experimental parameter, three independent sets of microalgal samples (10 mL each) were prepared to ensure reproducibility and statistical reliability. In addition, four parallel sets of identical samples were established for the assessment of cell growth.

All samples were incubated in a digital incubator shaker (71 L; Model 1–5311-DS-230 V, LABNET, USA) to maintain controlled and uniform operating conditions. The incubator was operated at a constant temperature of 30 ± 5 °C with a shaking speed of 120 rpm and continuous illumination at a light intensity of 1040 lx. The experimental setup was maintained for 96 h (4 days), during which biohydrogen production was monitored.

###  Data collection

The total volume of biogas produced and the hydrogen concentration were measured at 24 h intervals over a continuous period of 4 days. The cumulative gas volume was determined using a gas-tight syringe, while hydrogen concentration was quantified using a portable hydrogen gas detector (gas type: hydrogen; model: ATO-SKY2000-H2). These measurements were used to calculate the volume of hydrogen produced during the experimental period.

###  Determining the optimal parameters for biohydrogen production

A stepwise experimental strategy was adopted to systematically identify the optimal operating conditions for biohydrogen production. Each parameter was evaluated independently to isolate its individual effect on hydrogen yield.

Initially, nitrogen gas was purged into the test tubes for 2 min at a pressure of 0.5 bar, after which the vessels were immediately sealed with rubber stoppers to prevent oxygen ingress to compare hydrogen production under purged and non-purged conditions.

Following the identification of the condition yielding higher hydrogen production, the effect of photoperiod during the anaerobic phase was investigated by comparing three illumination regimes: continuous light (L/D 24:0), continuous darkness (L/D 0:24), and a light/dark cycle of 24 h light followed by 24 h dark (L/D 24:24). During the light phases of the photoperiod experiments, illumination was provided by the built-in cool white fluorescent lamps of the digital incubator shaker delivering a uniform light intensity of 1040 lx. Dark conditions were achieved by completely wrapping the test tubes with aluminum foil to prevent light penetration.

Subsequently, the influence of glucose supplementation as an additional carbon source was examined at concentrations of 5, 10, and 15 g L⁻^1^ to evaluate its impact on hydrogen production. Glucose stock solutions were prepared by dissolving the appropriate amount of D( +)-glucose monohydrate in distilled water to achieve the desired concentrations. All experiments were conducted in 10 mL test tubes containing 9 mL of the microalgal suspension and 1 mL of glucose solution prepared.

Finally, the effect of temperature was assessed by comparing hydrogen yields at 25, 30, and 35 °C. The digital incubator shaker was set to the respective temperatures while all other experimental conditions were maintained constant.

###  Cell growth analysis

As described in Sect. 2.5, four parallel samples were prepared for the cell growth analysis. To preserve anaerobic conditions throughout the hydrogen production phase, only one sample was opened at each sampling interval for OD measurement. Cell growth was monitored daily by measuring OD at 683 nm. The measured OD values were converted to cell number concentration using the calibration curve established in Sect. 2.2 relating OD to direct cell counts. The specific growth rate, µ (h^−1^), was calculated during the exponential growth phase according to Eq. (2) (Wahidin et al. 2013). The reported µ values represent the average of three independent replicates.

2 $$ \mu = \frac{{\ln \left( {N_{2} - N_{1} } \right)}}{{t_{2} - t_{2} }} $$

where *N*₁ and *N*₂ represent the cell number concentration (cells/mL) at times *t*₁ and *t*₂ (h), respectively.

###  Jacketed reactor scale-up

**Fig. 2:**
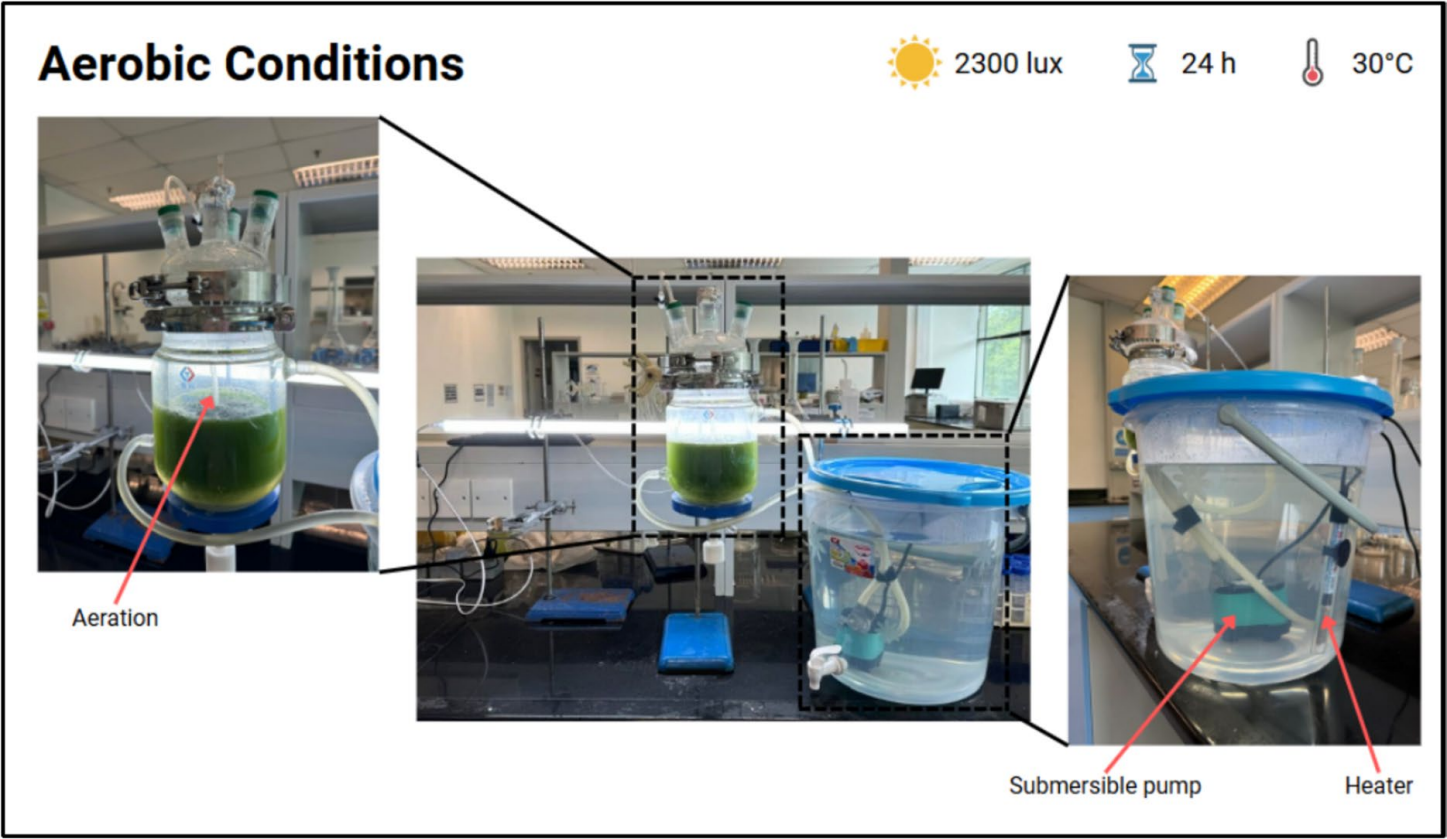
Schematic illustration of the jacketed reactor operated under aerobic conditions

Scale-up experiments were conducted under the previously optimized operating conditions using a 1000 mL jacketed glass reactor (Tengke, BORO 3.3). A schematic representation of the jacketed reactor setup is shown in Fig. 2. The reactor was operated at a working volume of approximately 800 mL, leaving sufficient headspace to facilitate gas accumulation and prevent excessive pressure build-up during cultivation.

Illumination was provided by a fluorescent light source at an intensity of 2300 lx, positioned using a retort stand to ensure uniform light exposure. The reactor jacket was filled with water and connected to an external water reservoir via rubber tubing. Temperature control was maintained at 30 °C using an aquarium heater, while a submersible water pump was employed to continuously circulate water through the jacket, thereby ensuring stable and uniform thermal conditions throughout the experiment.

The reactor was charged with a mixture consisting of 90% (v/v) microalgal culture and 10% (v/v) glucose solution. Prior to loading, 720 mL of the microalgal suspension was analyzed for optical density at 683 nm (OD₆₈₃) to ensure that the culture concentration was within the range specified in Sect. 2.4. Aeration was provided using an air pump equipped with a polytetrafluoroethylene (PTFE) filter (0.45 µm) to maintain sterility.

**Fig. 3:**
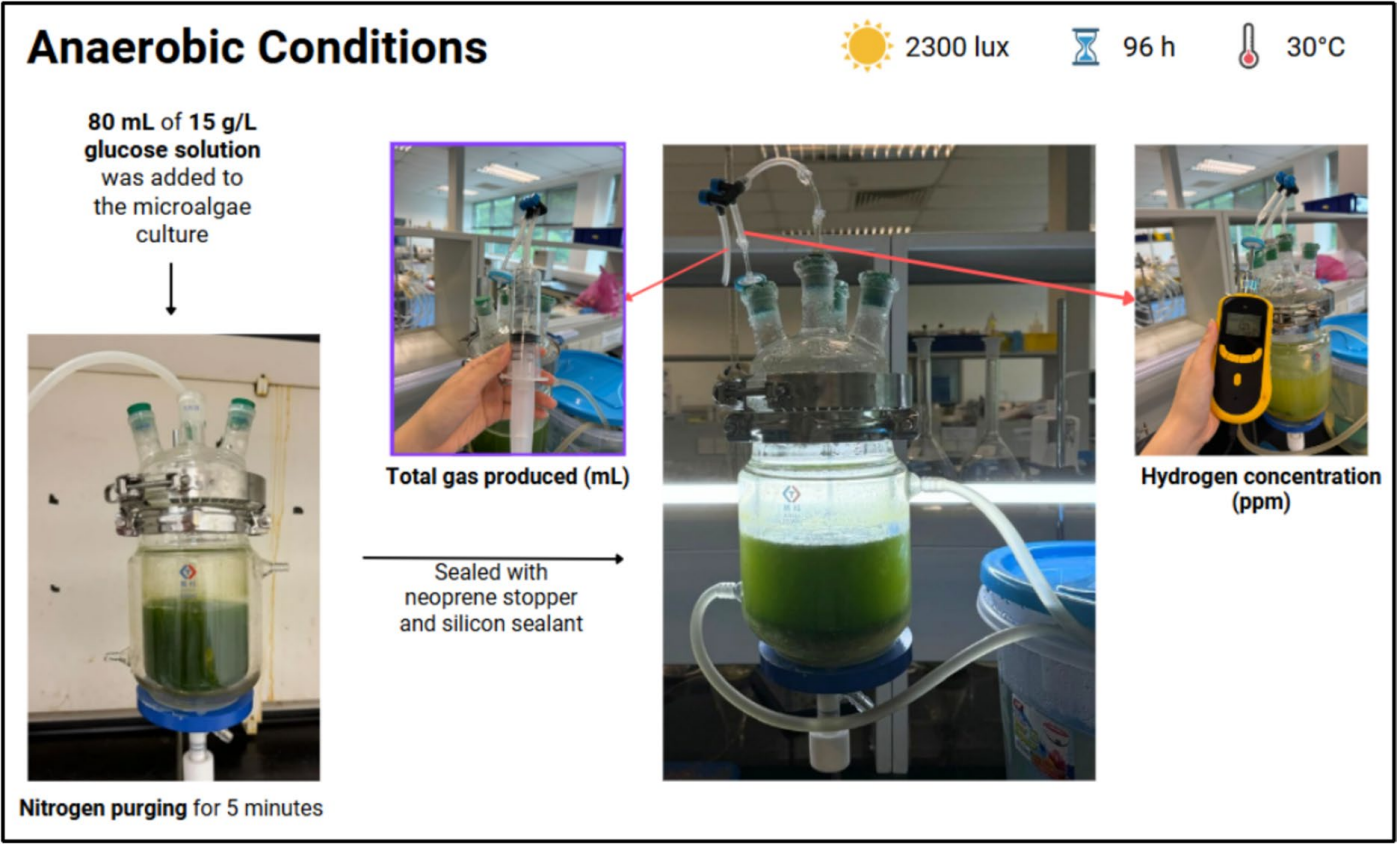
Schematic illustration of the jacketed reactor operated under anaerobic conditions

The culture was cultivated under continuous illumination and aeration for 24 h, after which OD₆₈₃ was measured again to monitor changes in biomass concentration. Continuous aeration with sparger also promotes mixing through bubbling to ensure periodic light exposure of cells and to reduce prolonged shading. Subsequently, 80 mL of a 15 g L⁻^1^ glucose solution was added to the reactor. Anaerobic conditions were then established by purging the reactor with nitrogen gas for 5 min, followed by immediate sealing with neoprene stoppers. A schematic representation of the jacketed reactor operated under anaerobic conditions at 30 °C is presented in Fig. 3. Gas measurements were conducted for 12 consecutive days to monitor hydrogen production over time. During the anaerobic phase, because no aeration is provided (i.e., no bubbling), the jacketed reactor is gently shaken manually every 2 h to promote mixing.

###  Microalgae biomass harvesting

Following the completion of biohydrogen production, microalgal biomass was harvested by refrigerated centrifugation. Culture samples were transferred into 50 mL centrifuge tubes and centrifuged at 4000 rpm for 4 min at 25 °C. After centrifugation, the supernatant was carefully decanted, and the resulting biomass pellets were collected.

The harvested microalgal biomass was subsequently frozen overnight and freeze-dried using a laboratory freeze dryer (Fisher, 1.5 L; Labconco) at − 40 °C for 48 h (Ramos et al. 2025). The dried biomass was then stored for future use.

###  Statistical analysis

All experiments were conducted in triplicate, and the results are presented as mean values. SigmaPlot version 15.0 (Systat Software, USA) was used for data processing and graphical representation.

## Results and discussion

###  Calibration curve

**Fig. 4:**
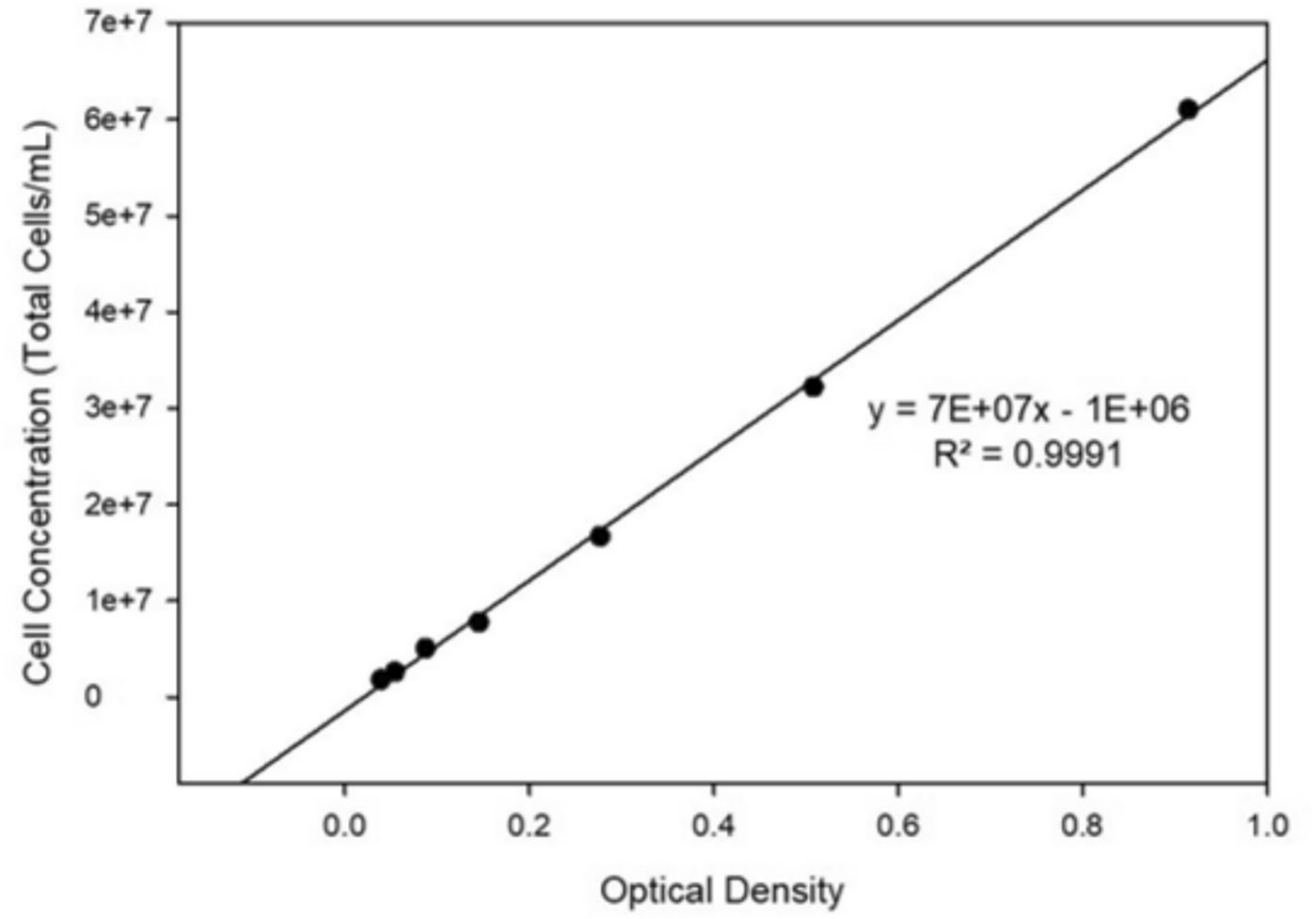
Calibration curve relating optical density to microalgal cell concentration

Figure 4 presents the calibration curve correlating optical density with microalgal cell concentration. The relationship between cell concentration and optical density is described by Eq. (3), exhibiting a high coefficient of determination (R^2^ = 0.9991), which confirms an excellent linear fit with negligible deviation across the measured range. The strong linear correlation indicates that optical density can be reliably used as a proxy for estimating microalgal biomass concentration during the experimental period.

3 $$ y = 7 \times 10^{7} x - 1 \times 10^{6} $$

where *y* represents the cell concentration (cells mL⁻^1^) and *x* denotes the optical density.

###  Effect of nitrogen purging

**Fig. 5:**
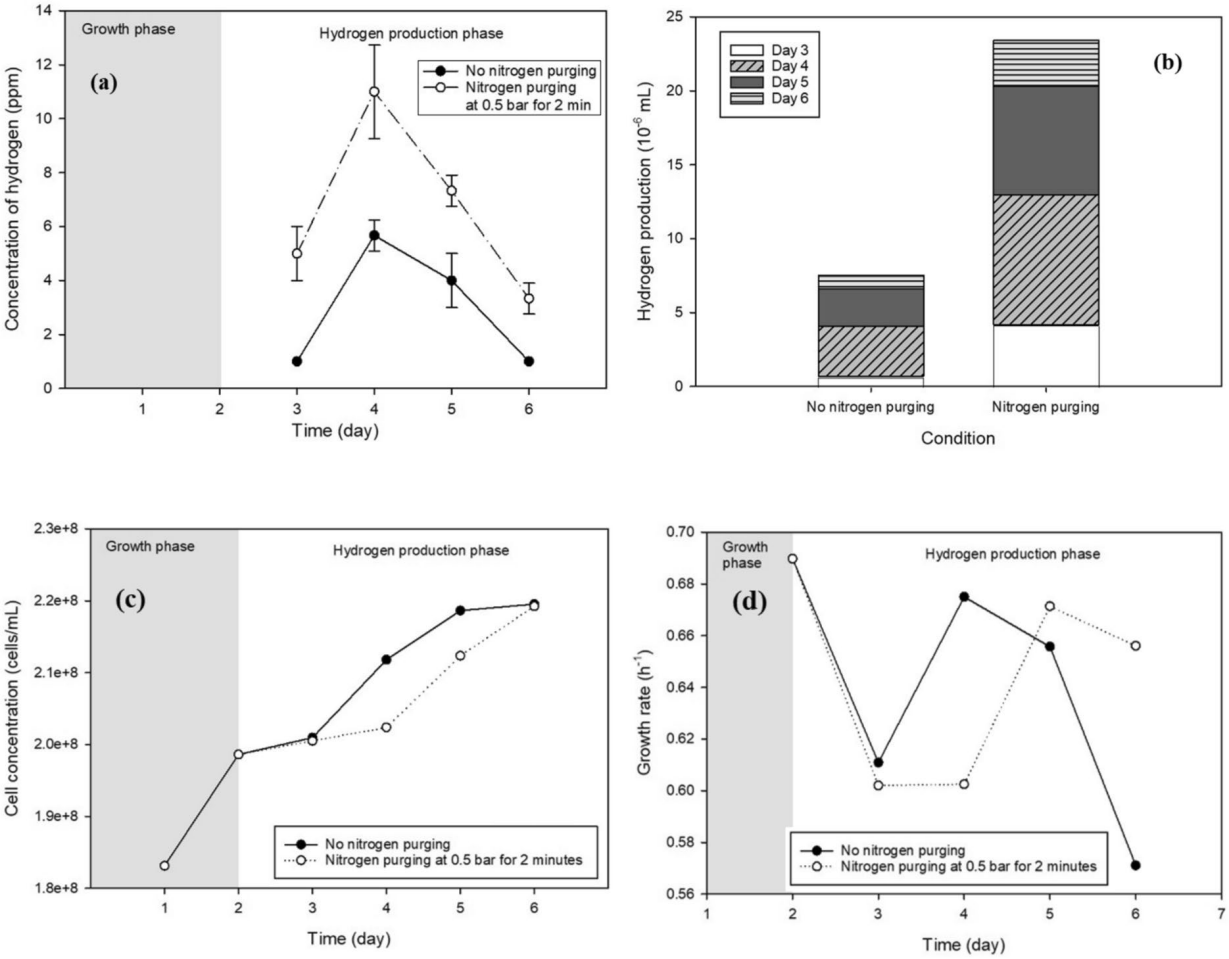
Effect of different purging conditions on **a** hydrogen concentration, **b** cumulative hydrogen volume, **c** cell concentration, and **d** cell growth rate

Hydrogen production experiments were conducted under fixed operating conditions, including an illumination intensity of 1040 lx, an agitation speed of 120 rpm, and an incubation temperature of 30 °C. The temporal profiles of hydrogen concentration and cumulative hydrogen production over the four-day anaerobic phase are presented in Fig. 5a, b, respectively. The low standard deviation values observed in Fig. 5b (ranging from 0 to 1.73) indicate good experimental reproducibility and measurement reliability.

In both purged and non-purged conditions, biohydrogen production increased following the induction of anaerobic conditions, reaching a maximum on the second day before subsequently declining. This trend is consistent with previous findings reported by Oncel et al. (2015), who demonstrated that peak hydrogen production in microalgal cultures typically occurs within 2–3 days after the establishment of anaerobic conditions. The decline observed after the peak can be attributed to nutrient depletion and the accumulation of inhibitory metabolic byproducts.

Nitrogen purging at 0.5 bar for 2 min resulted in a markedly higher hydrogen concentration compared to non-purged cultures. Under nitrogen-purged conditions, the hydrogen concentration reached a maximum of 11 ppm on the second day, whereas the non-purged cultures exhibited a lower peak of approximately 6 ppm at the same time point. Although hydrogen production decreased after reaching its maximum in both cases, nitrogen-purged cultures consistently exhibited higher hydrogen levels throughout the four-day production period. Despite this enhancement, the total volume of hydrogen collected remained relatively low, amounting to 7.53 × 10⁻⁶ mL and 2.34 × 10⁻^5^ mL for non-purged and nitrogen-purged conditions, respectively.

The enhancement of hydrogen production under nitrogen purging can be attributed to the establishment of anaerobic conditions, which are essential for activating hydrogenase enzymes. Hydrogenase is highly sensitive to oxygen, and even trace levels can significantly inhibit its activity in microalgae. These findings are in agreement with the work of Ananyev et al. (2008), who reported that nitrogen gas purging substantially enhanced hydrogen production in *Arthrospira* cultures by reducing dissolved oxygen levels and promoting sustained hydrogenase activity. In addition, Grechanik & Tsygankov (2022) reported that under anaerobic conditions, hydrogen production reaches its peak; however, as illumination continues, photosynthetic oxygen evolution leads to progressive inhibition of hydrogenase activity, resulting in a sharp decrease in hydrogen production.

The interaction between cell growth dynamics and hydrogen production is illustrated in Fig. 5c and Fig. 5d. In non-purged cultures, the presence of oxygen supported aerobic respiration, enabling higher ATP generation through complete oxidation of organic substrates and resulting in enhanced cell growth and biomass accumulation (Sarkar & Shimizu 2015)**.** Accordingly, non-purged cultures exhibited higher cell concentrations during the hydrogen production phase, as shown in Fig. 5c. In contrast, nitrogen-purged cultures displayed a slower growth rate, indicating that anaerobic conditions partially constrained cellular proliferation.

By day 6, the cell concentrations under nitrogen-purged and non-purged conditions were comparable (2.19 × 10⁸ and 2.20 × 10⁸ cells mL⁻^1^, respectively), demonstrating that *Chlorella* sp. can sustain growth under anaerobic stress, albeit at a slightly reduced rate. As the cultures transitioned into the hydrogen production phase, both cell concentration and specific growth rate (Fig. 5d) plateaued or declined, reflecting a metabolic shift driven by nutrient limitation and stress conditions.

Notably, the onset of the hydrogen production phase was marginally delayed in nitrogen-purged cultures (μ = 0.6020 h⁻^1^) compared to non-purged cultures (μ = 0.6109 h⁻^1^). This delay suggests that the abrupt transition to anaerobic conditions temporarily suppresses cell division. However, once acclimated, nitrogen-purged cultures redirected metabolic resources away from growth and toward hydrogen production, resulting in higher hydrogen yields, as evidenced in Fig. 5a.

Overall, these results demonstrate that biohydrogen production in *Chlorella* sp. is closely coupled with cellular growth phases. While the initial phase is dominated by biomass accumulation with minimal hydrogen evolution, the onset of stress conditions and anaerobiosis triggers a metabolic shift toward hydrogen production. This behavior supports the applicability of a two-stage (indirect) biophotolysis strategy for enhanced biohydrogen generation. Given the consistently higher hydrogen yields observed, nitrogen purging was selected as a critical operational step and applied in subsequent experimental stages.

###  Effect of photoperiod on biohydrogen production

**Fig. 6:**
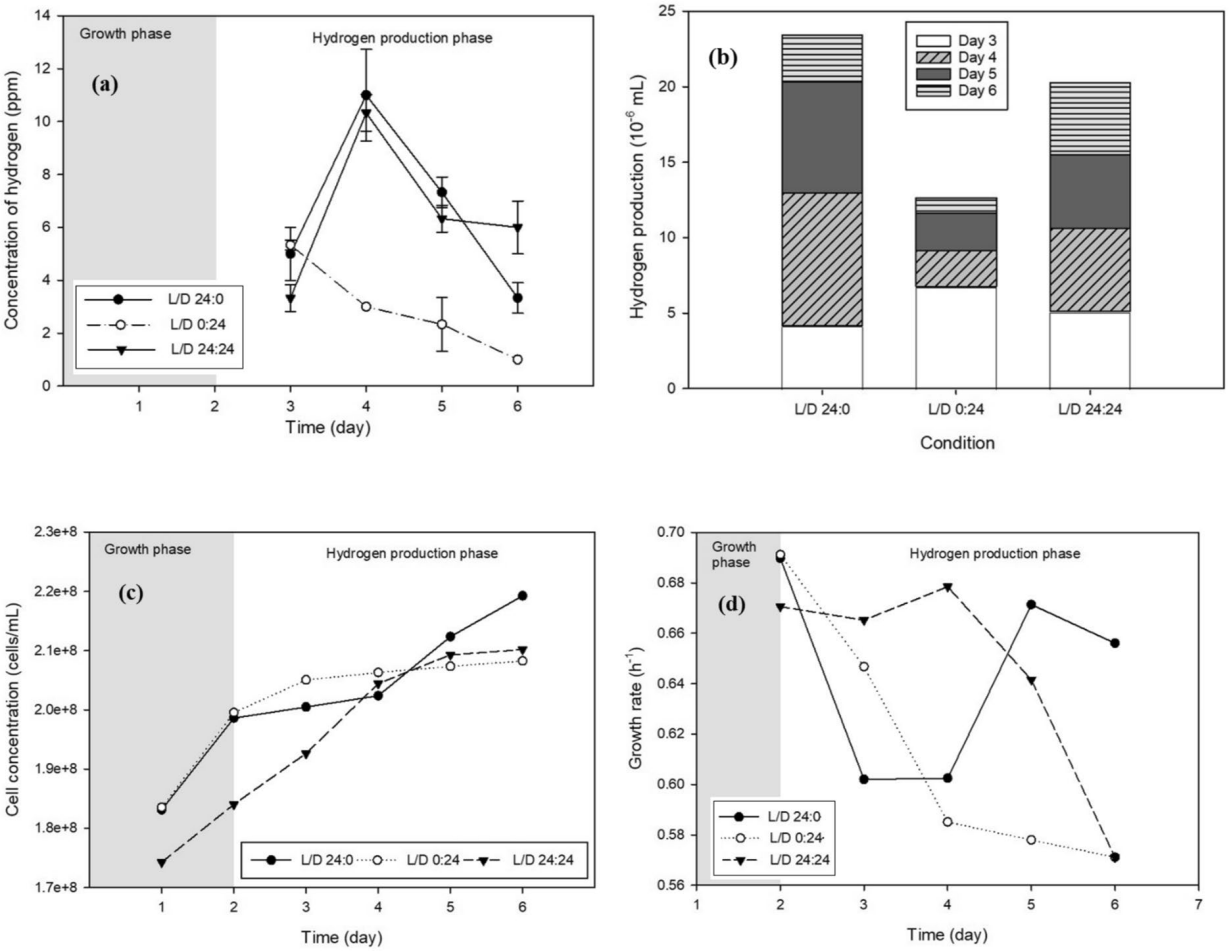
Effect of different photoperiods on **a** hydrogen concentration, **b** biohydrogen yield, **c** cell concentration, and **d** cell growth rate

Photoperiod experiments were conducted under fixed operating conditions, including nitrogen purging at 0.5 bar for 2 min, an agitation speed of 120 rpm, and an incubation temperature of 30 °C. Illumination was provided by a white fluorescent light source at an intensity of 1040 lx. The cumulative biohydrogen production under different photoperiod regimes is presented in Fig. 6b.

Among the tested conditions, continuous illumination (L/D 24:0) resulted in the highest hydrogen production yield (2.34 × 10⁻^5^ mL), followed by the alternating light/dark cycle (L/D 24:24; 2.03 × 10⁻^5^ mL). The lowest hydrogen yield was observed under continuous darkness (L/D 0:24; 1.26 × 10⁻^5^ mL). The superior performance of continuous illumination can be attributed to uninterrupted photosynthetic activity, which sustains electron flow and energy generation, thereby maintaining a favorable intracellular redox balance that supports hydrogenase activity (Antel et al. 2011).

The L/D 24:24 condition exhibited moderately lower hydrogen production compared to continuous light. As shown in Fig. 6a, peak hydrogen concentrations of 8.80 ppm and 5.51 ppm were observed on the second day for L/D 24:0 and L/D 24:24, respectively. The reduced yield under cyclic illumination is likely due to the interruption of photosynthesis during dark phases, which temporarily limits energy availability for hydrogen production. Nevertheless, alternating light and dark periods may contribute to the regulation of genes associated with hydrogen metabolism and mitigate oxygen accumulation, which is known to inhibit hydrogenase activity (Sanz-Luque et al. 2020).

Continuous darkness (L/D 0:24) resulted in the lowest hydrogen production among the tested photoperiods. Under dark conditions, hydrogen evolution relies primarily on fermentative metabolism, which is constrained by lower metabolic activity and limited availability of reduced electron carriers. Interestingly, Fig. 6a shows that transitions into dark phases (day 3 for L/D 0:24 and days 4 and 6 for L/D 24:24) coincided with transient increases in hydrogen production. This phenomenon can be explained by the cessation of photosynthetic oxygen evolution in the dark, followed by rapid oxygen consumption through respiration, thereby creating anaerobic conditions that favor hydrogenase activation (Catalanotti et al. 2013). During these periods, fermentative pathways generate reduced electron carriers such as NADH and reduced ferredoxin, which serve as electron donors for hydrogen production (Subramanian et al. 2014).

Cell concentration and specific growth rate trends under different photoperiods are illustrated in Fig. 6c, d. Continuous illumination (L/D 24:0) resulted in the highest cell concentration (2.19 × 10⁸ cells mL⁻^1^) and growth rate (0.6561 h⁻^1^) by day 6, reflecting enhanced biomass accumulation driven by sustained photosynthetic activity. The L/D 24:24 condition exhibited slightly lower biomass accumulation, as growth was periodically limited during dark phases. In contrast, continuous darkness (L/D 0:24) led to the lowest cell concentration and growth rate due to the absence of photosynthesis and reduced energy generation.

These observations are consistent with previous reports indicating that longer illumination periods promote higher biomass accumulation and intracellular carbon storage in microalgae (Oncel et al. 2015). In cyclic photoperiods, stored carbon compounds such as starch are partially consumed during dark phases to support cellular respiration, resulting in lower net biomass accumulation. Overall, continuous illumination simultaneously enhanced cell growth and hydrogen production, demonstrating its suitability for maximizing biohydrogen yield. Consequently, the L/D 24:0 photoperiod was selected and applied in subsequent experiments investigating the effect of carbon substrate addition.

###  Effect of glucose addition as carbon substrate

**Fig. 7:**
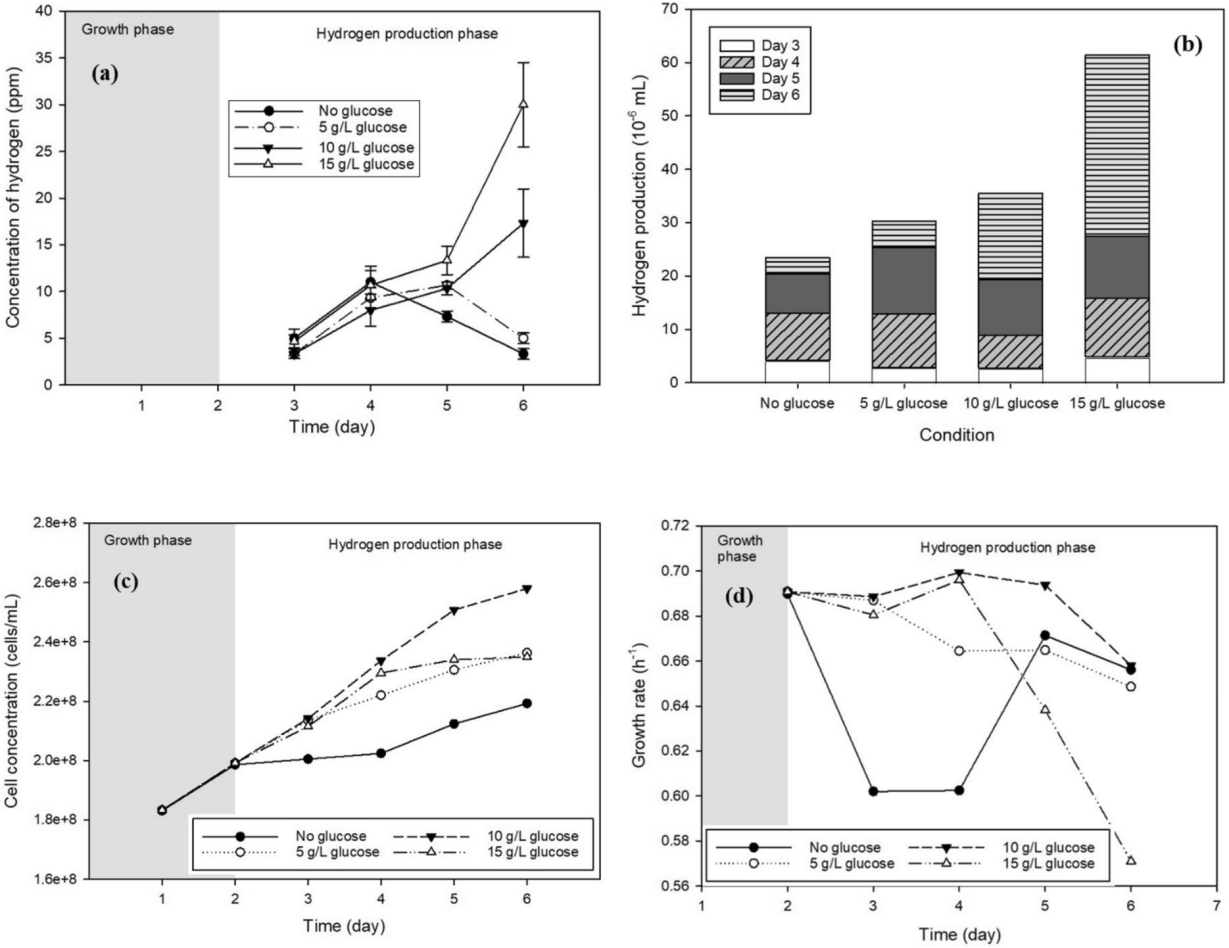
Effect of different glucose solution concentrations on **a** hydrogen concentration, **b** hydrogen yield, **c** cell concentration, and **d** cell growth rate

In this experimental stage, the operating conditions were fixed at nitrogen purging at 0.5 bar for 2 min per sample, continuous illumination (L/D 24:0) with a light intensity of 1040 lx, agitation at 120 rpm, and incubation at 30 °C. As illustrated in Fig. 7a, the supplementation of glucose significantly enhanced biohydrogen production in *Chlorella* sp. cultures, with hydrogen yield increasing proportionally with glucose concentration. Based on Fig. 7b, the highest cumulative hydrogen production was achieved at 15 g/L glucose (6.14 × 10⁻^5^ mL), followed by 10 g/L (3.54 × 10⁻^5^ mL), 5 g/L (3.03 × 10⁻^5^ mL), and the control without glucose (2.34 × 10⁻^5^ mL).

The observed enhancement in hydrogen production upon glucose addition can be attributed to multiple mechanisms. First, glucose suppied during the transition phase serves as an additional carbon and energy source, promoting the accumulation of intracellular energy reserves such as starch and lipids, which can subsequently be metabolized for hydrogen generation under anaerobic conditions (Li et al. 2024). Second, glucose availability stimulates key enzymes involved in hydrogen metabolism, particularly hydrogenases, thereby increasing hydrogen production rates. As shown in Fig. 7b, the 15 g/L glucose condition achieved a peak hydrogen concentration of 30 ppm on day 6, indicating that glucose not only increases total hydrogen yield but also sustains hydrogen production over an extended period.

Glucose supplementation also influenced the growth behavior of *Chlorella* sp., as reflected by cell concentration (Fig. 7c) and specific growth rate (Fig. 7d). Cell density increased with glucose addition, with the highest concentration observed at 10 g/L glucose (257,993,000 cells/mL), followed by 5 g/L (236,328,000 cells/mL), 15 g/L (234,928,000 cells/mL), and the no-glucose condition (219,255,000 cells/mL). This enhancement in biomass production can be attributed to the mixotrophic metabolic capability of *Chlorella* sp., which enables simultaneous utilization of organic carbon and photosynthetic energy. The presence of glucose accelerates cell division and biomass accumulation relative to photoautotrophic growth.

However, higher glucose concentrations also promote faster nutrient depletion and the accumulation of metabolic by-products, which may induce an earlier transition into the stationary phase. This behavior is particularly evident under the 15 g/L glucose condition (Fig. 7d), where growth deceleration coincides with elevated hydrogen production, suggesting a metabolic shift from biomass accumulation to hydrogen generation. These findings are consistent with previous reports.

Deng et al. (2019) observed that *Chlorella kessleri* exhibited a low specific growth rate (0.41 day⁻^1^) and maximum biomass of 0.31 g/L under photoautotrophic conditions after six days. In contrast, mixotrophic cultivation with 2–15 g/L glucose resulted in significantly higher growth rates (0.76–1.27 day⁻^1^) within the first two to three days and maximum biomass concentrations ranging from 1.05 to 4.46 g/L. Notably, biomass increased substantially up to 10 g/L glucose, while higher concentrations (12–15 g/L) yielded diminishing returns due to limitations in essential nutrients such as nitrogen and phosphorus. Similar saturation effects at elevated glucose levels have been reported by Cheirsilp and Torpee (2012).

Overall, glucose supplementation effectively supports the mixotrophic metabolism of *Chlorella* sp., providing additional carbon and energy for enhanced biomass formation and biohydrogen production. Although 15 g/L glucose did not result in the highest cell concentration, it was selected as the optimal condition in this study due to its superior biohydrogen yield, which represents the primary objective of the process.

###  Effect of temperature

In this experimental stage, the operating conditions were maintained as nitrogen purging at 0.5 bar for 2 min, continuous illumination (L/D 24:0) at 1040 lx, glucose supplementation at 15 g/L, agitation at 120 rpm, and incubation at the designated temperatures of 25, 30, and 35 °C.

**Fig. 8:**
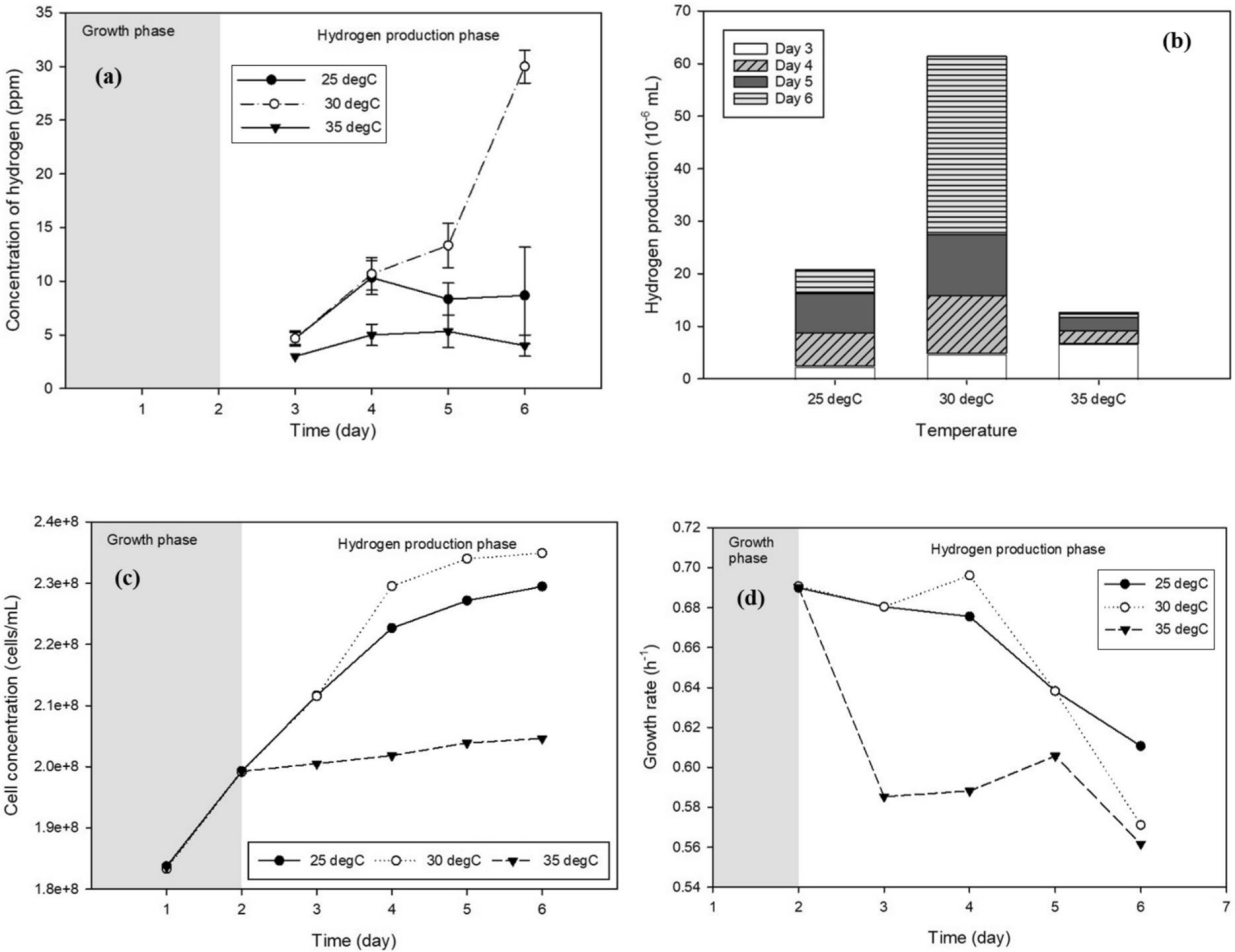
Effect of different temperatures on **a** hydrogen concentration, **b** hydrogen yield, **c** cell concentration, and **d** cell growth rate

The biohydrogen production profiles presented in Fig. 8a demonstrate that temperature plays a critical role in regulating hydrogen yield in *Chlorella* sp. cultures. Among the tested conditions, the highest cumulative hydrogen production was achieved at 30 °C, followed by 25 °C, while the lowest yield was observed at 35 °C. As shown in Fig. 8b, cultures incubated at 30 °C exhibited a rapid increase in hydrogen production during the hydrogen production phase, reaching a maximum yield of 6.14 × 10⁻^5^ mL. The 25 °C condition also supported substantial hydrogen generation, albeit at a lower level, with a total yield of 4.38 × 10⁻^5^ mL. In contrast, hydrogen production at 35 °C was markedly suppressed throughout the experimental period, resulting in a final yield of only 2.07 × 10⁻^5^ mL.

The superior hydrogen production at 30 °C can be attributed to an optimal balance between enzymatic activity and thermal stability, which facilitates efficient hydrogenase function and electron transfer processes. At this temperature, cellular metabolism remains sufficiently active to supply reducing equivalents for hydrogen evolution without imposing excessive thermal stress. Conversely, the reduced hydrogen yield observed at 35 °C suggests partial inhibition or destabilization of temperature-sensitive enzymes involved in hydrogen metabolism. This observation is consistent with the findings of Paramesh et al. (2018), who reported that although *Chlorella vulgaris* can tolerate elevated temperatures, deviations beyond the optimal range may impair metabolic efficiency. Furthermore, elevated temperatures can induce denaturation of critical metabolic enzymes and inactivation of photosynthetic proteins, while low temperatures coupled with carbon limitation inhibit photosynthesis, all of which disrupt cellular respiration and photosynthetic electron flow, ultimately reducing the efficiency of biohydrogen production by limiting the metabolic energy and electron supply required for sustained hydrogen synthesis (Jiao et al. 2024; Ekpan et al. 2025).

Temperature-dependent variations were also evident in cell concentration and growth rate, as shown in Fig. 8c, d, respectively. The highest cell concentration was recorded at 30 °C (234,928,000 cells/mL), followed closely by 25 °C (229,482,000 cells/mL), while the lowest biomass accumulation occurred at 35 °C (204,611,000 cells/mL). Across all temperatures, a decline in growth rate was observed upon entry into the hydrogen production phase, reflecting a metabolic shift from biomass formation to hydrogen generation.

At 25 °C, the growth rate decreased gradually from 0.6900 h⁻^1^ on day 2 to 0.6107 h⁻^1^ on day 6, maintaining the highest growth stability during the hydrogen production phase. In contrast, cultures incubated at 30 °C exhibited moderate fluctuations in growth rate, followed by a sharper decline to 0.5711 h⁻^1^, indicating a transition toward hydrogen-producing metabolism at the expense of cellular growth. The most pronounced instability was observed at 35 °C, where the growth rate declined sharply from 0.6903 h⁻^1^ on day 2 to 0.5616 h⁻^1^ on day 3, accompanied by subsequent fluctuations. This behavior suggests the onset of thermal stress, which may impair cellular homeostasis and reduce both growth and hydrogen production efficiency.

Overall, the results indicate that 30 °C represents the optimal temperature for *Chlorella* sp. under the tested conditions, providing a favorable compromise between robust cell growth and maximal biohydrogen production during the anaerobic phase. Accordingly, this temperature was selected as the optimal operating condition, and the final optimized parameters for biohydrogen production via the two-phase biophotolysis process are summarized in Table 1.

**Table 1:** Optimal conditions for biohydrogen production from microalgae

Purging conditions	Nitrogen purging at 0.5 bar for 2 min
Photoperiod	L/D 24:0; continuous illumination
Glucose concentration	15 g/L glucose solution for 10% of the photobioreactor operating volume
Temperature	30℃

### Jacketed reactor scale up

The optimal operating conditions summarized in Table 1 were applied to scale up the microalgae cultivation system from 10 mL test tubes to a 1000 mL jacketed reactor. The scale-up from small laboratory test tubes to a controlled jacketed photobioreactor is an important step toward translating microalgae-based biohydrogen production from proof-of-concept experiments into a more practical reactor platform. A substantial enhancement in hydrogen productivity was achieved after scale-up. The average hydrogen yield obtained from the 1000 mL jacketed reactor with an 800 mL working volume reached 6.22 × 10⁻^3^ mL H_2_/day, which corresponds to an approximately 405-fold increase compared to the yield obtained from 10 mL test tubes (1.535 × 10⁻^5^ mL H_2_/day) during the 12-day anaerobic hydrogen production phase. This pronounced increase demonstrates the strong influence of reactor scale and operational stability on biohydrogen production efficiency.

**Fig. 9:**
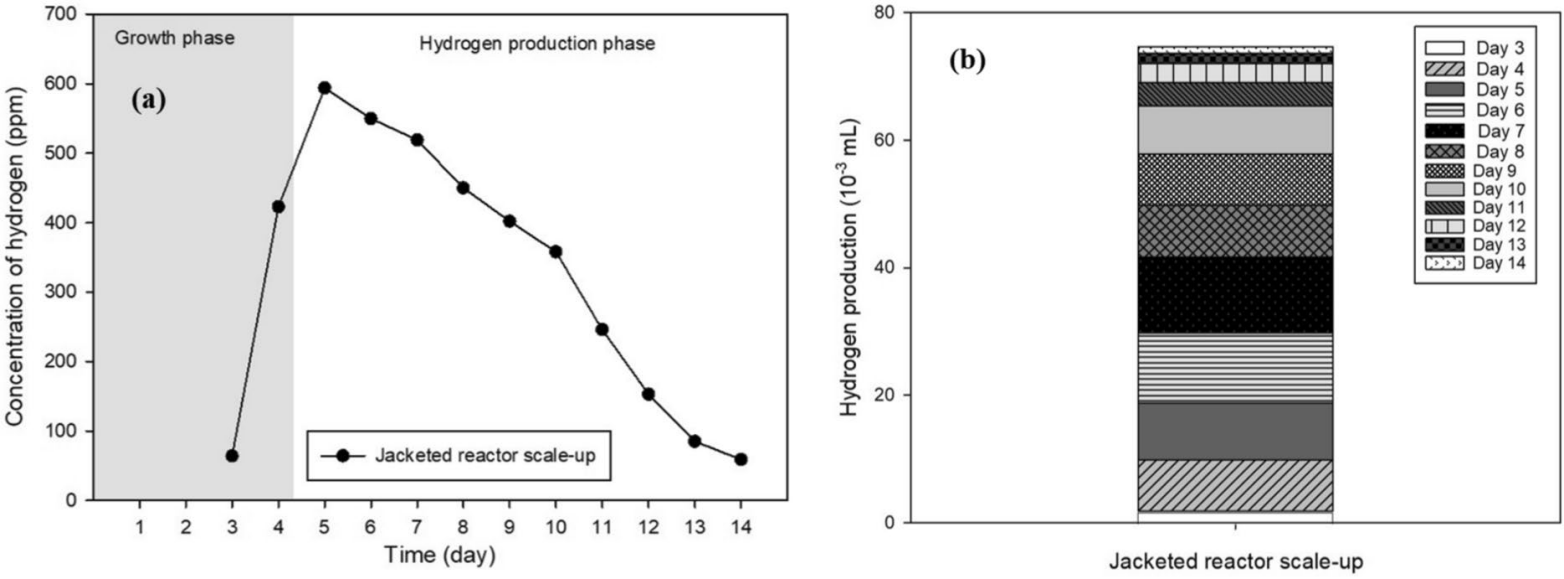
Hydrogen production performance obtained from the jacketed reactor: **a** hydrogen concentration and **b** hydrogen yield

As illustrated in Fig. 9a, the hydrogen concentration rapidly increased during the early stage of the anaerobic phase, reaching a maximum of 594 ppm on the second day, followed by a gradual decline to 59 ppm by day 14. The sharp initial increase indicates effective activation of hydrogenase enzymes under optimized anaerobic conditions, while the subsequent decline is likely attributable to substrate depletion, metabolic shifts, and accumulation of inhibitory byproducts over prolonged cultivation. These observations align with recent findings that temperature-modulated photosynthesis switching and oxygen scavenging strategies can extend hydrogenase activity, though further optimization of nutrient delivery and metabolic regulation remains necessary (Li et al. 2024).

Scaling up microalgal systems often leads to reduced performance due to challenges in maintaining consistent cultivation conditions (Hawrot-Paw & Ratomski 2024). Therefore, successfully translating optimized laboratory conditions into a controlled reactor system highlights an important contribution of this work. The enhanced hydrogen production observed at the larger scale can be attributed to improved parameter control, including more stable temperature regulation, enhanced mass transfer, and more uniform mixing compared to small-scale test tubes. Additionally, the increased culture volume allows for higher overall metabolic activity and more efficient utilization of available carbon and energy sources, thereby promoting sustained hydrogen production.

These findings highlight the potential of jacketed photobioreactors operated under optimized conditions for scalable biohydrogen production. If comparable growth and hydrogen production efficiencies can be maintained at larger reactor volumes, microalgae-based hydrogen generation could emerge as a viable renewable energy pathway. Nevertheless, several challenges must be addressed before further scale-up can be realized, including achieving uniform light distribution, effective heat management in larger volumes, and optimized nutrient delivery. Moreover, the energy requirements associated with illumination, agitation, and temperature control must be carefully balanced against hydrogen output to ensure overall process energy efficiency.

Overall, this study distinguishes itself from existing literature by not only optimizing key cultivation parameters but also successfully demonstrating their scalability in a controlled reactor system with significantly enhanced hydrogen production. Further research focusing on reactor design optimization, energy balance assessment, and long-term operational stability is still required to fully realize the potential of large-scale microalgae-based biohydrogen production systems.

## Conclusion

This study systematically investigated the influence of algal gorwth parameters on biohydrogen production and cell growth in *Chlorella* sp. cultures, employing a stepwise optimization strategy to enhance hydrogen yield through a two-stage (indirect) biophotolysis process.

The transition from aerobic to anaerobic conditions was identified as a critical phase for initiating hydrogen production. Nitrogen gas purging effectively established anaerobic conditions, leading to a marked enhancement in biohydrogen production compared to non-purged cultures. This improvement is attributed to the removal of dissolved oxygen, which activates oxygen-sensitive hydrogenase enzymes and induces a metabolic shift in *Chlorella* sp. towards hydrogen-producing pathways.

Photoperiod was found to significantly affect hydrogen production performance. Among the tested conditions, continuous illumination (L/D 24:0) resulted in the highest biohydrogen yield, followed by the alternating light–dark cycle (L/D 24:24), while continuous darkness (L/D 0:24) produced the lowest yield. Sustained illumination supports continuous photosynthesis, enabling the generation of reducing equivalents necessary for hydrogen metabolism and promoting overall hydrogen productivity.

The effects of carbon substrate supplementation and temperature during the anaerobic phase were also evaluated. Glucose addition significantly enhanced hydrogen production, with the highest yield achieved at a concentration of 15 g/L. The presence of an external organic carbon source supports mixotrophic metabolism, providing additional energy and carbon skeletons that facilitate sustained hydrogen production. Temperature optimization demonstrated that 30 °C is the most favorable condition for biohydrogen production, outperforming both 25 °C and 35 °C. This temperature provides an optimal balance between enzymatic activity and cellular stability, whereas higher temperatures may induce thermal stress and impair metabolic functions.

Evaluation of microalgal biomass growth revealed a close relationship between cell density, growth rate, and hydrogen production. A clear metabolic trade-off was observed, whereby increased hydrogen production coincided with a reduction in growth rate due to the redirection of cellular resources from biomass accumulation to hydrogen evolution. These findings underscore the importance of identifying an optimal balance between cell growth and hydrogen production to ensure process stability and sustained biohydrogen output.

Scale-up experiments conducted under the optimized conditions demonstrated a successful transition from 10 mL test tubes to a 1 L jacketed reactor, resulting in an approximately 405-fold increase in hydrogen yield. This substantial improvement highlights the feasibility and potential of scaling up microalgae-based biohydrogen production systems when appropriate operational conditions are applied.

Overall, this study emphasizes the critical role of algal growth parameters in regulating biohydrogen production in *Chlorella* sp. Although the findings are species-specific, the optimized conditions identified provide valuable insights for the development of efficient and sustainable microalgae-based biohydrogen production systems. The results support the viability of two-stage biophotolysis as a promising strategy for renewable hydrogen generation and offer a foundation for future scale-up and process optimization studies.

## Data Availability

No datasets were generated or analysed during the current study.
